# Molly Fancher’s Bazaar

**Published:** 1890-07

**Authors:** 


### MOLLY FANCHER'S BAZAAR.

We have heretofore given some account of Molly Fancher, that marvel of a bed-ridden invalid, who, for more than twenty years, has been a helpless cripple, in our sister city of Brooklyn.

In 1867, Molly was one of the most promising pupils of the well known Packer Institute. A serious accident deprived her of the use of her limbs, and reduced her to a state of almost utter helplessness, from which she has never recovered. During all these years of prostration, Miss Fancher has continued to support herself by various means. For a long period her arms were rigidly held upward, her hands meeting at the back of her head, and whilst so held at a point which precluded the aid of her natural sight, she wrought a variety of fancy work of exquisite design and finish. Her patience under affliction is something marvellous. As if in compensation for the impairment of her physical organs, Miss Fancher is endowed with wonderful faculties of perception and prevision. She seems to be aware of every thing that is taking place about her. With a mind ever alert, she is able to devise means and methods with remarkable clearness. Her latest venture is a Bazaar at 160 Gates avenue, Brooklyn, directed by her by means of a speaking tube, connected with her couch on an upper floor.

The invalid directs her two assistants, keeps the books, even to the slightest detail, and watches the progress of her enterprise.

For months at a time she does not take food of any kind, and really seems, as she lies wan and wasted upon her accustomed bed, less a mortal than a spirit. The case is a remarkable one, and so far as we are informed, unprecedented.

